# The effectiveness of an ultra‐brief intervention in 1 min for hazardous drinking in a general hospital setting: A quasi‐randomized pilot trial

**DOI:** 10.1002/pcn5.216

**Published:** 2024-06-18

**Authors:** Yukio Tezuka, Ryuhei So, Takahiro Fukuda

**Affiliations:** ^1^ Okinawa Rehabilitation Center Hospital; ^2^ Okinawa Chubu Hospital; ^3^ Okayama Psychiatric Medical Center Okayama Japan; ^4^ CureApp Inc.; ^5^ Scientific Research WorkS Peer Support Group (SRWS‐PSG); ^6^ Akiyama Hospital

**Keywords:** alcohol‐related disorders, alcohol use disorders, hazardous drinking, screening and brief intervention, ultra‐brief intervention

## Abstract

**Objective:**

We investigated the effectiveness of an ultra‐brief intervention (Ultra‐BI) for patients with hazardous drinking behaviors admitted to a general hospital.

**Method:**

In a quasi‐randomized controlled trial at a general hospital in Japan, we assigned participants to intervention or control groups based on the last digit of their patient ID (odd for intervention, even for control). The study included inpatients with Alcohol Use Disorder Identification Test‐Consumption (AUDIT‐C) scores of ≥5 for men and ≥4 for women. The intervention involved providing advice and feedback within 1 min, accompanied by a leaflet on alcohol‐related issues (Ultra‐BI). The control group did not receive any intervention. The primary outcome was average weekly alcohol consumption at 3 months postintervention.

**Results:**

The study included 68 participants. The intervention group showed a reduction in average weekly alcohol consumption by −69.7 g/week compared to the control group (95% confidence interval [CI] −145.7 to 6.3 g/week, *p* = 0.07). Post‐hoc analysis, adjusting for baseline values, indicated a between‐group difference of −78.7 g/week (95% CI −135.2 to −22.2 g/week, *p* = 0.007).

**Conclusion:**

This pilot trial suggests the potential effectiveness of the Ultra‐BI in general hospital wards. Further large‐scale studies are required to confirm these findings.

## INTRODUCTION

The global impact of alcohol is substantial, with one in seven deaths attributed to its consumption.^1^ Excessive alcohol consumption is a significant risk factor for more than 200 diseases and injuries.^2^ Medical institutions treating such conditions are likely to encounter numerous patients with a history of excessive alcohol consumption. Studies in general hospitals have found that approximately 20% of male and 5% of female inpatients engage in excessive alcohol use.^3^, ^4^

Addressing excessive alcohol use in general hospitals is crucial for preventing the recurrence and exacerbation of alcohol‐related diseases. Screening and brief intervention (SBI) is a known effective measure in this setting that consists of identifying individuals with hazardous drinking behavior and engaging them in short conversations aimed at modifying their drinking behavior.^5^ SBI for 15–60 min has not only been shown to reduce alcohol intake, but also to decrease mortality rates at 6 and 12 months, as evidenced by a meta‐analysis.^6^ Moreover, improvement in mental health following SBI has been reported in a randomized controlled trial (RCT).^7^

However, excessive alcohol consumption in general hospitals has not been sufficiently addressed. Research has indicated that medical staff can identify only 25.4% of cases involving alcohol abuse, and their recognition rate of alcohol dependence is approximately 30%.^8^ Even after introducing SBI for alcohol use in three UK hospital wards, screenings were only conducted in 18%–37% of patients during the observation period.^9^ The most common barriers to implementing SBI cited by healthcare providers are cost and time.^10^

A closer examination of previous studies revealed an ongoing debate regarding the effectiveness of time‐saving interventions, such as providing information alone compared with more time‐intensive counseling. An RCT in a general hospital found that 20 min of counseling and providing a specialized booklet on alcohol issues resulted in comparable reductions in alcohol consumption after 6 months.^11^ Another RCT reported that a group receiving 20 min of face‐to‐face counseling demonstrated a greater reduction in alcohol consumption after 6 months compared to those who received a general health information leaflet.^12^

In response to this controversy, our team developed an ultra‐brief intervention (Ultra‐BI) in which physicians handed out a leaflet specifically addressing alcohol‐related problems and provided supportive feedback messages in 1 min. The Ultra‐BI is designed as a feasible and cost‐effective strategy in busy clinical settings. We conducted a quasi‐RCT in the inpatient wards of a Japanese general hospital to investigate the effectiveness of our Ultra‐BI in patients with excessive alcohol use.

## METHODS

### Trial design

This was a single‐center quasi‐RCT.

### Settings and procedures

We recruited participants from patients admitted to all clinical departments at Okinawa Chubu Hospital on July 19, 2019. The tertiary medical center in Okinawa Prefecture, Japan, has 559 beds and 40 clinical departments and primarily focuses on acute care. To screen patients eligible for hazardous drinking, we used the first three items of the Alcohol Use Disorders Identification Test (AUDIT),^13^, ^14^ which is called AUDIT‐C.^15^

### Randomization, concealment, and blinding

We predetermined to allocate eligible patients with an odd last digit of their patient ID to the intervention group and those with an even last digit to the control group. Although this procedure was regarded as quasi‐randomization, it was difficult to predict who would be admitted to the hospital on the day of the baseline survey (July 19, 2019) at the time of submission of the study protocol to the institutional review board for ethical review (June 10, 2019). Given this condition, we considered that allocation concealment would be maintained. We did not blind the participants or the researcher who performed eligibility checks, allocation, intervention, and assessment of outcomes based on responses from the participants because of the following procedure of the baseline survey. In the survey, medical staff in each ward distributed an informed consent document and a questionnaire that included the AUDIT. Subsequently, one of the researchers (Y. T.) visited all the wards to collect the written informed consent forms and calculate the AUDIT‐C scores as an eligibility check. For patients who met the eligibility criteria, the researcher provided the Ultra‐BI (intervention group) or simply expressed gratitude for the patient completing the questionnaire (control group).

### Participants

The inclusion criteria were inpatients aged 18–75 years and a score of AUDIT‐C ≥5 for men and ≥4 for women.^16^ The exclusion criteria were patients admitted to the intensive care unit, high care unit, or maternal–fetal intensive care unit; patients incapable of providing written consent or understanding the baseline survey questionnaire; and patients whose primary physician or medical staff judged them unsuitable for this trial.

### The intervention and the control conditions

In the intervention group, the researcher, who had specialized in addiction treatment for 3 years and developed the leaflet for the Ultra‐BI, handed out a double‐sided A4‐sized leaflet (Figure S1) and provided feedback and some comments within 1 min as follows: “Mr./Ms. [Name], you might drink too much (Feedback). I recommend you to calculate your alcohol consumption on your own (Advice). The information here (in the leaflet) will be beneficial for you (Guarantee of effectiveness). You can easily apply these recommendations starting today (Assurance of feasibility). I look forward to hearing your thoughts when we meet next time (Motivation through commitment).” Neither any leaflet nor feedback was provided to the control group.

### Data collection

In the baseline survey, we conducted a 15‐item assessment that included the 10 items based on the AUDIT. If there was missing information in the participants' responses, the researchers confirmed the details with the participants at the time of obtaining the response sheet. For the second AUDIT question, which required detailed input regarding the type of beverage, container type, and quantity, the researchers calculated the total volume of pure alcohol to determine the specific score for this question. Additionally, during data collection, the first three questions of the AUDIT‐C were scored to ascertain the eligibility of participants for the study.

Three months after the baseline survey and intervention, the researcher mailed a follow‐up questionnaire to the participants. The questionnaire comprised five questions: (1) frequency of drinking (five options based on the first question of AUDIT‐C), (2) quantity of alcohol consumed per occasion (equivalent to the second question of AUDIT‐C), (3) number of heavy drinking days (five options based on the third question of AUDIT‐C), (4) a question regarding whether they received advice about drinking during admission (three options: received, not received, do not remember), and (5) thoughts about the intervention (three options: welcomed, not welcomed but acceptable, wish to stop). If there were any missing responses in the questionnaire, the researcher followed up with the participants directly via phone to confirm their answers.

Regarding the frequency of drinking (Question 1), we made approximations based on the responses. A score of 0 points, indicating no drinking, was equated to 0 times per week. For a score of 1 point, corresponding to drinking once a month or less, the frequency was approximated as 0.5 times per month or 0.125 times per week. A score of 2 points, denoting drinking two to four times a month, was calculated to represent three times per month, or 0.75 times per week. A score of 3 points, representing drinking two to three times per week, was interpreted as 2.5 times per week. Finally, a score of 4 points, reflecting drinking four to seven times per week, was assessed for an average of 5.5 times per week.

Regarding the quantity of alcohol consumed, the participants reported the types of beverages, alcohol content, types of containers used, and number of units consumed. If the participants drank multiple types of beverages, they were instructed to list up to three types separately. If some required information was missing, we contacted the participants via phone for clarification. If we were unable to contact them, we imputed them as follows:

We used the following typical alcohol content values for each beverage: beer was assigned a standard alcohol content of 5%; sake (Japanese rice wine) 15%; *shochu*/*awamori* (Japanese spirits) 25%; wine 12%; cocktails made with Japanese spirits 5%; and whiskey, brandy, tequila, gin, and other distilled spirits 40%. The typical content values were determined after the responses were collected.

To determine the volume of each type of beverage, we set the following container sizes: for beer, the volumes were set at 350 mL for a can, 500 mL for a bottle, 350 mL for a mug, and 180 mL for a cup. The volumes were determined at 180 mL for a glass and 30 mL for a small cup (an *ochoko*). The serving size for *shochu*/*awamori* was standardized at 180 mL. For wine, we considered 120 mL for a glass and 720 mL for a bottle. Cocktails made with Japanese spirits were assigned a volume of 350 mL per can. Finally, for whiskey, brandy, tequila, gin, and other spirits, the standard serving size was set at 30 mL.

Using these imputed and converted values, we calculated the quantity of alcohol consumed on each occasion. We then converted this into the corresponding option for the second question of the AUDIT.

### Outcomes

The primary outcome was the average weekly alcohol consumption (g/week). To calculate this, we multiplied the average alcohol consumption (g/week) by the weekly frequency of alcohol consumption. The secondary outcomes were the AUDIT‐C total score and the AUDIT‐C score for each question.

### Statistical method

We used EZR for all statistical analyses. EZR is statistical software that provides a graphical user interface for R.^17^ For the baseline data, we calculated descriptive statistics of the demographics and characteristics of the participants according to the allocated group. For the primary outcome data, we computed the point estimates and 95% confidence intervals (95% CIs) of between‐group differences and tested the statistical significance using a *t*‐test. Additionally, we conducted a post‐hoc linear regression analysis to estimate the between‐group analysis, adjusting for the baseline value of average alcohol consumption. For secondary outcomes, the score of each of the three items and the total score of the AUDIT‐C, we tested the statistical significance of the between‐group differences.

### Sample size

We did not determine the sample size in advance. As this was a pilot study, we recruited as many participants as possible on a single day, as conducted by a single researcher.

### Ethical considerations

We conducted this study after receiving approval from the Ethics Review Committee of Okinawa Chubu Hospital. Written consent was obtained from all participants for their involvement in the study.

## RESULTS

### Participant flow and follow‐up

**Figure 1:**
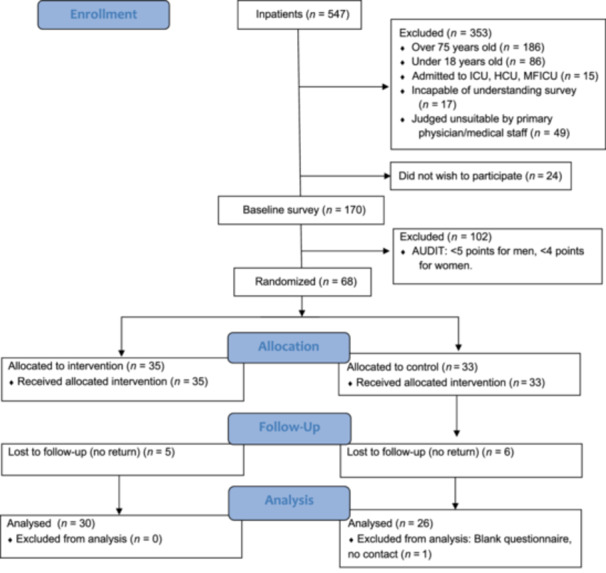
The CONSORT flow diagram. HCU, high care unit; ICU, intensive care unit; MFICU, maternal–fetal intensive care unit.

As depicted in Figure 1, of the 194 individuals who met the eligibility criteria for the baseline survey, 170 (87.6%) consented to participate. Based on the AUDIT‐C scores, we included 68 patients in the quasi‐randomized trial, with 35 allocated to the intervention group and 33 to the control group. In the follow‐up survey, 30 participants from the intervention group and 26 from the control group returned their responses (56/68, 82%). We could not include one participant in the control group in the outcome data analysis because the returned questionnaire from that participant was almost completely blank and we were unable to contact them subsequently.

### Baseline data

Table 1 presents the background information of the participants. There were no statistically significant differences in the background characteristics between the intervention and control groups. The participants tended to be middle‐aged. Approximately one‐fifth of the participants had AUDIT scores ≥15, suggesting a potential risk of alcohol dependence. The baseline average alcohol consumption was greater in the intervention group by 15.5 g/week compared to the control group.

**Table 1:** Baseline characteristics of participants (*n* = 68)

	Intervention group	Control group	
	*n* = 35	*n* = 33	*p*‐value^a^
Age (years)	50.6	51.2	0.864
Gender (male %)	18 (51.4)	21 (63.6)	0.337
Average AUDIT score	10.6	11.1	0.744
Average AUDIT‐C score	6.89	7.00	0.828
Score for AUDIT Question 1	3.00	2.97	0.914
Score for AUDIT Question 2	2.20	2.27	0.809
Score for AUDIT Question 3	1.69	1.76	0.825
AUDIT score above 15 (%)	7 (20)	8 (24.2)	0.773
AUDIT score above 8 (%)	23 (65.7)	21 (63.6)	1
Average alcohol consumption (g/week)	204.86	189.36	0.678

Abbreviation: AUDIT, Alcohol Use Disorder Identification Test; AUDIT‐C, AUDIT‐Consumption (the first three items of the AUDIT).

^a^ *t*‐test.

### Outcomes

**Figure 2:**
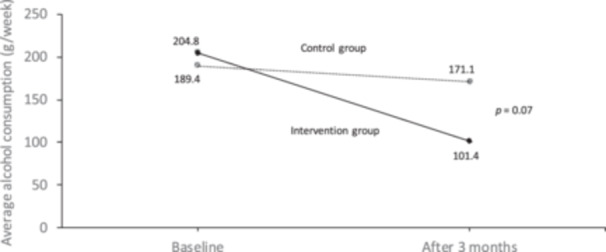
Changes in average alcohol consumption.

The primary outcome, average alcohol consumption at 3 months, was 101.4 g/week in the intervention group and 171.1 g/week in the control group. The between‐group difference in the primary outcome was −69.7 g/week (95% CI –145.7 to 6.3 g/week, *p* = 0.07) (Figure 2). Based on the post‐hoc regression analysis adjusting for baseline value, the estimated between‐group difference was −78.7 g/week (95%CI −135.2 to −22.2 g/week, *p* = 0.007).

The secondary outcome, the AUDIT‐C score at 3 months postintervention, was 3.97 for the intervention group and 6.08 for the control group, showing a statistically significant decrease in the intervention group (Table 2). In the analysis of individual AUDIT items, the quantity of alcohol consumed per occasion (AUDIT Question 2) and the number of heavy drinking days (AUDIT Question 3) significantly decreased in the intervention group, whereas there was no significant difference in drinking frequency (AUDIT Question 1).

**Table 2:** AUDIT‐C scores at 3 months postintervention.

	Intervention group	Control group	*p* value^a^
AUDIT‐C	3.97	6.08	0.009
AUDIT Question 1	2.30	2.73	0.25
AUDIT Question 2	1.10	1.84	0.028
AUDIT Question 3	0.56	1.50	0.004

Abbreviation: AUDIT, Alcohol Use Disorder Identification Test; AUDIT‐C, AUDIT‐Consumption (the first three items of the AUDIT).

^a^ *t*‐test.

Of the 30 participants in the intervention group, 21 (70%) remembered receiving the intervention, three (10%) reported not receiving it, and six (20%) could not recall. Of the 21 participants who remembered the intervention, 15 (71%) welcomed it, six (29%) did not welcome it but found it acceptable, and none expressed a negative attitude toward the Ultra‐BI.

## DISCUSSION

We examined the effectiveness of the Ultra‐BI in 1 min for patients admitted to a general hospital engaging in hazardous drinking using a quasi‐RCT design. As shown in Figure 1, the intervention group had a lower point estimate for average weekly alcohol consumption at 3 months compared to the control group, which underwent only assessment, although the difference was not statistically significant (−69.7 g/week, *p* = 0.07). However, the post‐hoc analysis adjusting for baseline values showed that average weekly alcohol consumption at 3 months in the intervention group was −78.7 g/week lower than in the control group (*p* = 0.007). Furthermore, none of the participants in the intervention group showed a negative attitude towards the Ultra‐BI.

Our results support the findings of previous studies suggesting the effectiveness of an Ultra‐BI without detailed advice or counseling. In general hospitals, Holloway et al. showed that a self‐help booklet reduced alcohol consumption by 80.0 g/week (95% CI 128 to 31.2 g/week) after 6 months compared to usual care.^11^ Although the between‐group difference of 69.7 g/week at 3 months in our study was not statistically significant, it aligned closely with the results of Holloway et al. Also in a general hospital, Freyer‐Adam et al. found that computer‐generated feedback letters were as effective as 35 min of in‐person counseling in reducing alcohol consumption over 2 years.^7^ In primary care settings, Kaner conducted a three‐arm cluster RCT comparing simple feedback and a patient information leaflet, 5 min of advice, and 20 min of counseling, but found no significant differences in AUDIT scores at 6‐month follow‐up. These findings imply that time‐saving interventions to provide well‐crafted written materials and simple feedback, such as an Ultra‐BI, can be as effective as more time‐consuming BI approaches.

The potential effectiveness of the Ultra‐BI may have been enhanced by the general hospital setting. A trial conducted in an emergency department showed no significant effects on leaflet distribution.^18^ Another trial found no impact of mailing leaflets to the general population compared to an assessment‐only control group.^19^ In contrast to these populations, our hospitalized patients, who were experiencing health issues and had established trust with the medical staff, might have been more receptive to receiving advice and feedback on their drinking habits and were possibly more ready to reduce alcohol consumption. Furthermore, having sufficient time to engage with the material may have contributed to its effectiveness. The positive attitude towards the Ultra‐BI further supports its appropriateness and potential effectiveness in the hospital ward context. Additionally, the process of our study proved the feasibility of implementing the Ultra‐BI in busy clinical settings, as a single researcher was able to screen 170 inpatients and provide the intervention to 35 of them in a single day.

Our study had five major limitations. First, we approximated drinking frequency based on the response to Item 1 of the AUDIT‐C to calculate the weekly average alcohol consumption. This may have compromised the precision in estimating the differences between the groups.

Second, the randomization process based on patient numbers did not allow for concealment. However, we predetermined that odd numbers would comprise the intervention group in the research‐planning stage. Given the unpredictability of hospital admissions, it was impossible to manipulate patient numbers to favor the researchers. The high participation rate (89% of those meeting the inclusion criteria) and balanced distribution of participants across groups indirectly suggest a low likelihood of bias in the recruitment process.

Third, the generalizability of our findings is limited. It was conducted in a single facility with a small sample size, and the interventions were administered by an experienced principal investigator. Whether similar results would be obtained in other regions or with different researchers is uncertain. However, as a pilot study, these encouraging results provide a rationale for conducting larger‐scale, long‐term confirmatory trials.

Fourth, the follow‐up period of 3 months was relatively short. Previous studies have reported that differences between groups are more pronounced or sustained at 6 or 12 months compared to 4 months.^20^ Whether this pattern holds true for our intervention and population warrants further investigation.

Finally, our study did not collect data to explore the mechanism of action of the Ultra‐BI. However, detailed assessments for this purpose may themselves influence the drinking behavior of participants and could potentially mask the effects of BIs that are thought to have small effect sizes. Therefore, to determine which elements of the Ultra‐BI are effective, methods that do not increase the amount of assessment for participants, such as component network meta‐analysis of RCTs, may be needed.^21^

## CONCLUSIONS

We demonstrated the potential efficacy of the Ultra‐BI in patients admitted to a general hospital. The low cost and feasible nature of the Ultra‐BI suggests that its integration into routine clinical practice could effectively reduce alcohol consumption among people engaging in hazardous drinking. Our preliminary but promising results justify conducting a multisite RCT to confirm the effectiveness of the Ultra‐BI and its generalizability in general hospital settings.

## DECLARATION OF GENERATIVE AI IN SCIENTIFIC WRITING

During the preparation of this work, the authors used ChatGPT and Grammarly in order to proofread and polish up sentences. After using these tools, the authors reviewed and edited the content as needed and took full responsibility for the content of the publication.

## AUTHOR CONTRIBUTIONS

**Yukio Tezuka:** Conceptualization; data curation; formal analysis; investigation; methodology; project administration; resources; visualization; writing—original draft; writing—review and editing. **Ryuhei So:** Writing—original draft; writing—review and editing; supervision. **Takahiro Fukuda:** Conceptualization; methodology; writing—review and editing.

## CONFLICT OF INTEREST STATEMENT

Dr. Tezuka reports personal fees from Otsuka Pharmaceutical Co., MSD K.K. Dr. So reports personal fees from CureApp Inc., during the conduct of the study; grants from Osake‐no‐Kagaku Foundation, The Mental Health Okamoto Memorial Foundation, and Kobayashi Magobe Memorial Medical Foundation, and personal fees from Otsuka Pharmaceutical Co., Ltd, Nippon Shinyaku Co., Ltd, and Takeda Pharmaceutical Co., Ltd outside the submitted work. In addition, Dr. So has a patent JP2022049590A, US20220084673A1, JP2022178215A, JP2022070086, and a patent JP2023074128A pending. Dr. Fukuda does not report any conflict of interest.

## ETHICS APPROVAL STATEMENT

We conducted this study after receiving approval from the Ethics Review Committee of Okinawa Chubu Hospital.

## PATIENT CONSENT STATEMENT

Written consent was obtained from all participants for their involvement in the study.

## CLINICAL TRIAL REGISTRATION

https://jrct.niph.go.jp (jRCT1090220426).

## Supporting information

Figure S1. The leaflet used for the ultra‐brief intervention (two pages printed on a single A4 sheet, double‐sided): (1) alcohol intake conversion chart, (2) assessment of the risks associated with alcohol consumption, (3) explanation of the benefits of reducing alcohol intake, (4) goal setting, (5) practical strategies for reducing alcohol intake, and (6) introduction to alcohol intake tracking tools.

## Data Availability

The participants of this study did not give written consent for their data to be shared publicly, so due to the sensitive nature of the research, supporting data is not available.
