# Supplementary material for: The effectiveness of an ultra‐brief intervention in 1 min for hazardous drinking in a general hospital setting: A quasi‐randomized pilot trial
Source: PCN Rep. 2024 Jun 18;3(2):e216. doi: 10.1002/pcn5.216 (PMC11187907; doi:10.1002/pcn5.216)

**sFigure 1. The leaflet used for the Ultra-brief intervention (two pages printed on a single A4 sheet, double-sided)** (1) alcohol intake conversion chart, (2) assessment of the risks associated with alcohol consumption, (3) explanation of the benefits of reducing alcohol intake, (4) goal setting, (5) practical strategies for reducing alcohol intake, and (6) introduction to alcohol intake tracking tools.
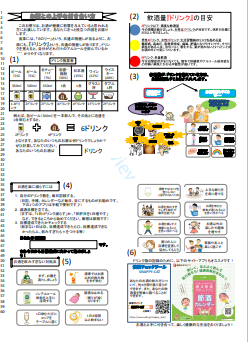

Supplement: Supplementary file 1 — Figure S1. The leaflet used for the ultra‐brief intervention (two pages printed on a single A4 sheet, double‐sided): (1) alcohol intake conversion chart, (2) assessment of the risks associated with alcohol consumption, (3) explanation of the benefits of reducing alcohol intake, (4) goal setting, (5) practical strategies for reducing alcohol intake, and (6) introduction to alcohol intake tracking tools. [file PCN5-3-e216-s001.docx]
